# CD4^+^ T cell counts and soluble programmed death-1 at baseline correlated with hepatitis B surface antigen decline in HIV/HBV coinfection during combined antiretroviral therapy

**DOI:** 10.3389/fcimb.2023.1178788

**Published:** 2023-05-03

**Authors:** Xiaodi Li, Ling Xu, Lianfeng Lu, Xiaosheng Liu, Yang Yang, Yuanni Wu, Yang Han, Xiaoxia Li, Yanling Li, Xiaojing Song, Wei Cao, Taisheng Li

**Affiliations:** ^1^ Department of Infectious Diseases, Peking Union Medical College Hospital, Peking Union Medical College and Chinese Academy of Medical Sciences, Beijing, China; ^2^ Department of Infectious Diseases and Clinical Microbiology, Beijing Chao-yang Hospital, Capital Medical University, Beijing, China; ^3^ Tsinghua-Peking Center for Life Sciences, Department of Basic Medical Sciences, School of Medicine, Tsinghua University, Beijing, China; ^4^ State Key Laboratory of Complex Severe and Rare Diseases, Peking Union Medical College Hospital, Chinese Academy of Medical Science and Peking Union Medical College, Beijing, China

**Keywords:** coinfection, HIV, HBV, HBsAg decline, soluble PD-1, immune activation

## Abstract

**Background:**

Several studies have described the rapid decline and clearance of hepatitis B surface antigen (HBsAg) in human immunodeficiency virus (HIV)/hepatitis B virus (HBV) coinfection after initiating combined antiretroviral therapy (cART). Early decline of HBsAg levels is associated with HBsAg seroclearance in the treatment of chronic HBV infection. This study aims to evaluate the HBsAg kinetics and the determinants of early HBsAg decline in patients with HIV/HBV coinfection during cART.

**Methods:**

A total of 51 patients with HIV/HBV coinfection were enrolled from a previously established HIV/AIDS cohort and followed for a median of 59.5 months after cART initiation. Biochemical tests, virology and immunology assessments were measured longitudinally. The kinetics of HBsAg during cART were analyzed. Soluble programmed death-1 (sPD-1) levels and immune activation markers (CD38 and HLA-DR) were measured at baseline, 1-year and 3-year during treatment. HBsAg response was defined as a decline of more than 0.5 log_10_ IU/ml at 6 months from the baseline after initiation of cART.

**Results:**

HBsAg declined faster (0.47 log_10_ IU/mL) in the first six months and attained a decrease of 1.39 log_10_ IU/mL after 5-year therapy. Seventeen (33.3%) participants achieved a decline of more than 0.5 log_10_ IU/ml at the first 6 months of cART(HBsAg response) of which five patients achieved HBsAg clearance at a median of 11 months (range: 6-51 months). Multivariate logistic analysis showed the lower baseline CD4^+^ T cell levels (OR=6.633, *P*=0.012) and sPD-1 level (OR=5.389, *P*=0.038) were independently associated with HBsAg response after cART initiation. The alanine aminotransferase abnormality rate and HLA-DR expression were significantly higher in patients who achieved HBsAg response than in those who did not achieve HBsAg response after cART initiation.

**Conclusion:**

Lower CD4 ^+^ T cells, sPD-1, and immune activation were related to a rapid HBsAg decline in patients with HIV/HBV-coinfection after the initiation of cART. These findings imply that immune disorders induced by HIV infection may disrupt immune tolerance to HBV, leading to a faster decline in HBsAg levels during coinfection.

## 1 Introduction

Human immunodeficiency virus (HIV) has affected an estimated 38.4 million people and caused approximately 650,000 deaths in 2021 (World Health Organization, 2022a). As a threat to worldwide public health, there were 296 million people infected with chronic hepatitis B infection (HBV) (World Health Organization, 2022b). People with HIV (PWH) are susceptible to HBV infection owing to similar transmission routes, and approximately 8.4% of PWH are coinfected with the hepatitis B virus (Leumi et al., 2020). In China, the coinfection of HIV/HBV is highly prevalent because of the high regional burden of HBV (Xie et al., 2016).

Hepatitis B surface antigen (HBsAg) seroclearance is considered the endpoint in chronic hepatitis B (CHB) functional cure studies; however, it is only achieved in few patients (Lok et al., 2012; Hsu et al., 2021; Hsu et al., 2022). Early significant decreases in serum HBsAg levels during treatment are related to HBsAg loss and can be used as a surrogate endpoint in exploratory studies on HBV functional cure (Cornberg et al., 2017; Gane et al., 2019; Cornberg et al., 2020). In Pegylated-interferon-α (PEG-IFNα) therapy, a decline in HBsAg levels at 12 or 24 weeks could predict an effective and sustained anti-HBV response (Sonneveld et al., 2010; Li et al., 2015; Boglione et al., 2016). HBsAg decline during nucleos(t)ide analog (NAs) therapy is considerably slower than that in PEG-IFNα therapy (Reijnders et al., 2011; Marcellin et al., 2016; Mak et al., 2023). During NAs treatment for HBV, a decrease in HBsAg level of more than 0.5 log10 IU/ml is considered a significant reduction, which is achieved within the first year of treatment in less than 10% of patients, and in 30% of patients within 2 years of treatment (Fung et al., 2011; Papatheodoridis et al., 2014; Papatheodoridis et al., 2015). HBsAg decline >0.5 log10 IU/ml in the first 6 months can lead to HBsAg levels of <100 IU/ml or HBsAg loss during NAs therapy (Wursthorn et al., 2010; Jeng et al., 2016; Liaw, 2019).

The immunoinhibitory receptor programmed death-1 (PD-1) combined with programmed death-ligand 1(PD-L1), can directly contribute to T-cell dysfunction and exhaustion. PD-1 expression on T cell is persistently upregulated during HIV and HBV infection (Day et al., 2006; Boni et al., 2007; Zhang et al., 2007). The soluble PD-1 (sPD-1) is an alternatively spliced mRNA transcript of exon 3, which is considered an inhibitor of the PD-1/PD-L1 pathway (Gu et al., 2018). Recent studies have indicated that sPD-1 could predict disease progression in CHB, given its association with an elevated risk of both liver fibrosis and hepatocellular carcinoma (Cheng et al., 2014; Zhou et al., 2019). Furthermore, Hu et al. found that a lower sPD-1 level could independently predict spontaneous HBsAg seroclearance in inactive CHB patients with undetectable HBV DNA (Chu et al., 2022; Hu et al., 2022). Given the similar immunological functions of PD-1 in chronic viral infections, we hypothesized that sPD-1 might also be associated with HBsAg clearance during HIV/HBV coinfection. However, limited information exists on sPD-1 profiles of patients with HIV/HBV coinfection.

Recently, several studies have reported a high prevalence of HBsAg seroclearance in HIV/HBV coinfection during combined antiretroviral therapy (cART) (van Bremen et al., 2020; Yoshikawa et al., 2021). Nevertheless, limited studies exist on the decline in HBsAg and its driving factors in HIV/HBV coinfection. In this study, we enrolled 51 patients with HIV/HBV coinfection from a previously established longitudinal HIV/acquired immunodeficiency syndrome (AIDS) cohort and extracted their clinical data for analysis. This study aimed to determine the association of hepatitis B e-antigen (HBeAg), baseline HBsAg level, sPD-1 and CD4 ^+^ T cell with HBsAg decline in HIV/HBV coinfection after the initiation of cART. Our study also ascertained determinants with a significant HBsAg decline(>0.5 log10 IU/ml) in early treatment in patients with HIV/HBV-coinfected.

## 2 Methods

### 2.1 Study population and design

The study participants were recruited from a longitudinal HIV/AIDS cohort conducted at the Peking Union Medical School Hospital (PUMCH). The inclusion criteria for the HIV/AIDS cohort have been described previously (Lu et al., 2022), including (1) HIV-1 infection diagnosed by detection of HIV-1 antibodies using western blotting, (2) not receiving cART, and (3) age 18–65 years. After enrollment, patients were followed up once every 3–6 months, during which routine clinical examinations and blood sample collection were conducted. The participants initiated cART based on the recommendations of the guidelines at that time. Medical and clinical data were recorded and uploaded to the PUMCH database.

**Figure 1:**
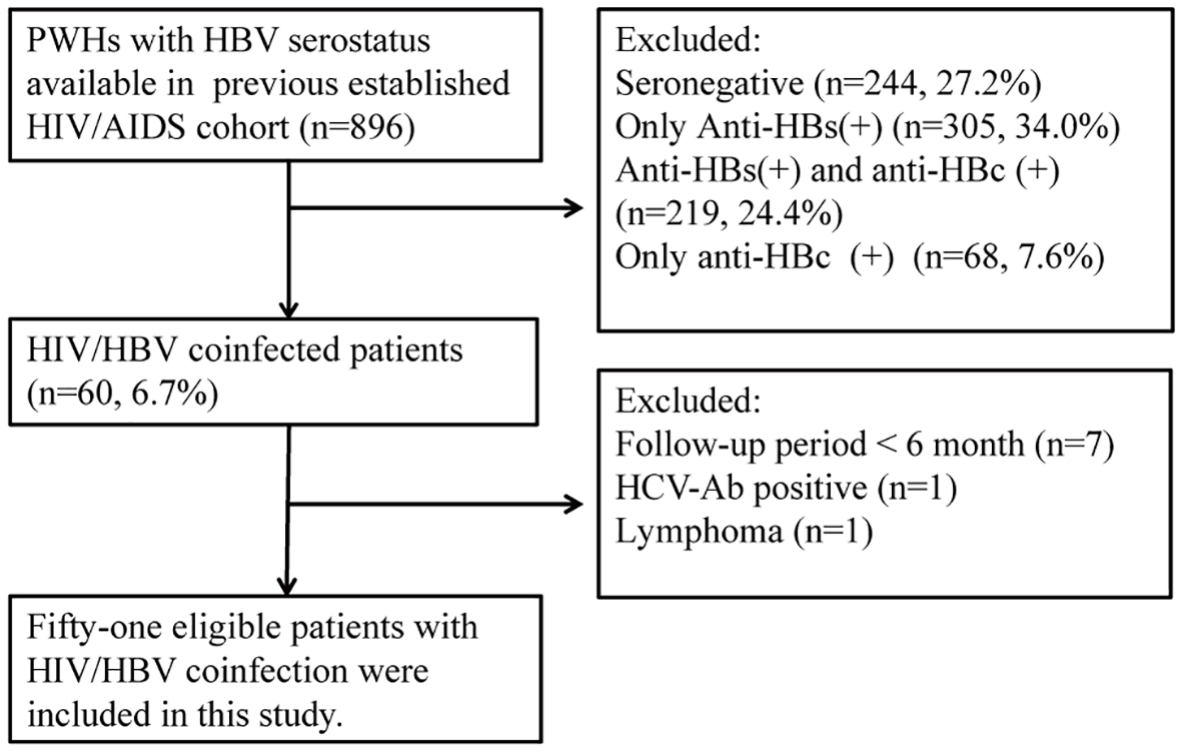
Study flowchart. PWH, people living with HIV.

We recruited patients with HIV/HBV coinfection from the HIV/AIDS cohort. The inclusion criteria of this study were as follows: (1) positive HBsAg or detectable HBV DNA lasting more than 6 months (2) no previous history of anti-HBV treatment. (3) patients who received cART for more than 6 months in the HIV/AIDS cohorts. The exclusion criteria were as follows: (1) coinfection with hepatitis C, (2) existence of alcoholic and autoimmune liver diseases, (3) history of malignancy, and (4) use of corticosteroids, immunosuppressants, or chemotherapeutic drugs in the past 6 months. Overall, 896 adult patients were screened from the HIV/AIDS cohort, and 51 eligible patients were enrolled in this study (**Figure 1**). This study retrieved clinical data before cART and each visit until the last follow. The cutoff date for data analysis was July 31, 2021.

This study was conducted following the Declaration of Helsinki and was approved by the Institutional Review Board of PUMCH. Written informed consent was obtained from each participant.

### 2.2 Laboratory tests

Routine liver function tests were performed at PUMCH laboratory. HBV serological markers (HBsAg, anti-HBs, HBeAg, anti-HBe, and HBcAg) were determined using the Abbott Architect platform(Abbott Diagnostics, USA). HBV viral load was quantified using the COBAS Ampliprep/TaqMan48 real-time polymerase chain reaction (PCR) Test (Roche Diagnostics, USA). Furthermore, HBV genotype was determined using PCR for the HBV reverse transcriptase gene. HIV-1 RNA levels were measured using the COBAS HIV-1 assay (Roche Diagnostics, USA). Flow cytometry was employed to evaluate CD4^+^ and CD8^+^ T cell counts and the presence of CD38 and HLA-DR markers (BD Biosciences, USA).

### 2.3 Quantification of HBsAg

HBsAg was quantified at baseline and 6, 12, 24, 36, 48 and 60 months in patients after the initiation of cART. Quantification of HBsAg was measured from frozen stored samples (-80°C) using the Abbott Architect HBsAg assay (Abbott Diagnostics, USA), with a range of 0.05–124,925 IU/ml.

### 2.4 Measurement of serum sPD-1

Serum sPD-1 levels were measured using a commercial ELISA Kit (Quantikine Elisa kit, R&D, USA), with a detection range of 15.6–1000 pg/ml. The sensitivity was 3.27 pg/ml. The intra-assay and inter-assay CV% were <10%. Serum was diluted appropriately when the concentration was outside this range. sPD-1 was quantified in participants at baseline, 1 year, and 3 years after cART.

### 2.5 Clinical definition

HBsAg response was described as a decline of more than 0.5 log_10_ IU/ml at 6 months from the baseline, which is a significant decline in patients with CHB under NAs therapy (Wursthorn et al., 2010; Liaw, 2019). To determine the factors correlated to HBsAg decline in HIV/HBV coinfection, coinfected participants were classified into the HBsAg response group (sAg-R group) and HBsAg non-response group (sAg-NR group) according to whether patients achieved HBsAg response. The upper limit of normal (ULN) for alanine aminotransferase (ALT) was defined as 19 U/L and 30 U/L in females and males, respectively (Terrault et al., 2016; Kao et al., 2020). The ULN for aspartate aminotransferase (AST) level was defined as 40 U/L. Liver fibrosis scores, including the AST-to-platelet(PLT) ratio index and fibrosis-4, were calculated as previously described (Maponga et al., 2020).

### 2.6 Statistical analysis

Serum levels of sPD-1 at baseline were categorized into three groups according to four quartiles: Q4: >908pg/mL, Q1: <417 pg/mL and below and above the median (Q2 and Q3: 417‐908pg/mL). Continuous variables were expressed as mean [standard deviation] or median (25th-75th percentile). Linear mixed-effects modeling was performed to investigate the HBsAg decline across the measured time points. A main effect of group (HBeAg positive vs negative; HBsAg <1000 IU/mL vs HBsAg≥1000 IU/ml at baseline; CD4 <200 cell/mm^3^ vs CD4 ≥200 cell/mm^3^; sPD-1<Q1:417pg/mL vs sPD-1 Q2–Q3: 417–908 pg/mL vs. sPD-1: Q4 908 pg/mL, respectively for individual model), a main effect of time (categorically coded for baseline, 6, 12, 24, 36, 48, and 60 months), and interactions between group * time were included in each model. Differences in continuous variables between the HBsAg response and non-response groups were analyzed using the independent samples t-test, and Mann–Whitney U test for normally and non-normally distributed data, respectively. Categorical variables were analyzed using the chi-square test or Fisher’s exact test. Furthermore, binary logistic regression was performed to determine the influence of characteristics on the HBsAg response. Variables with a P<0.10 in the univariate regression were included in multivariate logistic regression analyses. The multivariate logistic regression analyses were fitted based on maximum likelihood estimation. Statistical analysis was performed using SPSS 23.0 and GraphPad Prism 8.0.

## 3 Results

### 3.1 Participants characteristics

Fifty-one patients were enrolled in this study, and cART was initiated between July 2007 and November 2020. The characteristics of the participants with HIV/HBV-coinfection are presented in **Table 1**. At baseline, patients with HIV/HBV coinfection had a mean age of 35.2 years old, and most were men (92.2%). Twenty-one patients were tested for HBV genotypes, with 15 and 6 infected with HBV genotypes C and B, respectively. The median HBV-DNA levels were 2.70 log_10_ IU/ml. The HBsAg level was 3.82 log_10_ IU/ml. Twenty (40.8%) patients had elevated ALT levels. Regarding HIV infection-related parameters at baseline, the median HIV-RNA was at 4.53 log_10_ copies/ml. The mean CD4^+^ T cell was 227 cell/mm^3^, the mean CD8^+^ T cell was 830 cell/mm^3^, and the median CD4^+^/CD8^+^ ratio was 0.29. Patients had received cART for 59.5 (36.0–96.3) months. Forty-one (80.3%) patients initiated tenofovir disoproxil fumarate (TDF)+lamivudine (3TC)-based HBV therapy cART schedules, nine (17.6%) used 3TC, and one received tenofovir alafenamide fumarate/emtricitabine. After a median of approximately 5-year treatments, patients with HIV/HBV coinfection achieved HBV viral control, decreased HBsAg levels, decreased fibrosis score, and increased platelet counts. After treatment, the HIV viral load was controlled, and CD4^+^ T cell counts and CD4^+^/CD8^+^ ratio recovered significantly. Generally, these patients were successfully treated.

**Table 1:** The demographic and clinical characteristics of participants at baseline and the last visit.

Characteristic	HIV/HBV coinfection		p-value
	Baseline (n=51)	After Treatment (n=51)	
Age	35.2 (7.7)	40.6 (8.5)	–
Sex (% male)	47 (92.2)	47 (92.2)	–
HBeAg positive(%)	19 (37.3)	16 (31.4)	0.532
HBsAg level, log_10_ IU/mL	3.82 (2.95-4.54)	2.86 (2.09-3.77)	<0.001
HBV DNA level, log_10_ IU/mL	2.70 (1.45-7.98)	0 (0-0)	<0.001
ALT(U/L)	26 (19-42)	22 (17-35)	0.112
Elevated ALT above ULN(%)	20 (40.8)	16 (32.0)	0.362
AST(U/L)	26 (23-38)	22 (19-27)	<0.001
Elevated AST above ULN (%)	10 (20.4)	4(8.0 )	0.076
Total bilirubin, μmol/L	12.37 (4.88)	12.30 (5.43)	0.571
Platelet count, x10^9^/L	179 (47)	214 (61)	<0.001
APRI score	0.41 (0.30-0.55)	0.20 (0.20-0.37)	<0.001
FIB-4 score	1.19 (0.77-1.78)	0.89 (0.65-1.22)	0.001
HIV-RNA, log_10_ copies/ml	4.53 (3.39-4.96)	0 (0-0)	<0.001
CD4 T cell	227 (120)	512 (193)	<0.001
CD4 T cell <200 cell/mm^3^	18 (35.3%)	2 (3.9%)	<0.001
CD8 T cell	830 (398)	665 (300)	0.010
CD4/CD8 Ratio	0.29 (0.17-0.44)	0.81 (0.59-1.16)	<0.001

Data are presented as mean(standard deviation), number of patients(percent) or median(Interquartile range). Differences in variables were analyzed using the Paired-Samples T-Test, χ2 tests, and Wilcoxon matched-paired signed-ranked test, respectively.

HIV, human immunodeficiency virus; HBV, hepatitis B virus; HBeAg, hepatitis B envelope antigen; HBsAg, hepatitis B surface antigen; ALT, alanine aminotransferase; AST, aspartate aminotransferase; APRI, AST to Platelet Ratio index; FBI-4, Fibrosis-4. Data analyzed by the Wilcoxon matched-pair rank test.

### 3.2 Kinetics of HBsAg decline

**Figure 2:**
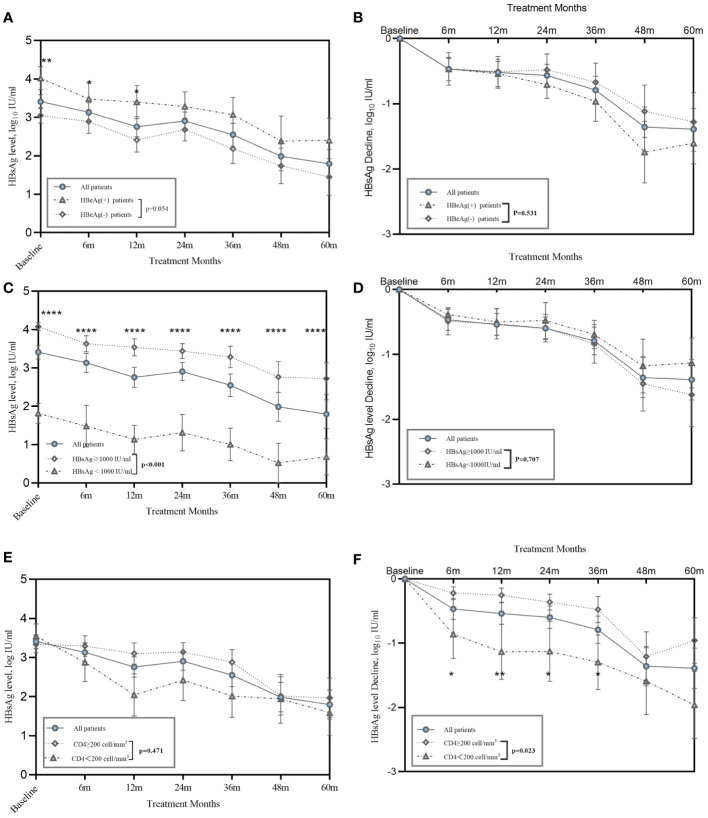
Kinetics of HBsAg in HIV/HBV coinfection patients during treatment. **(A)** Line graphs show mean ± SEM of HBsAg level, and **(B)** HBsAg decline in HIV/HBV coinfected patients stratified on baseline HBeAg status; **(C)** Line graphs show mean ± SEM of serum HBsAg level shows serum HBsAg level, and **(D)** HBsAg decline in HIV/HBV coinfected patients stratified on baseline HBsAg<1000 IU/ml or not; **(E)** Line graphs show mean ± SEM of serum HBsAg level, and **(F)** HBsAg decline in HIV/HBV coinfected patients stratified on baseline CD4^+^<200 cells/mm^3^ or not. *P*-values were determined using a linear mixed-effects model. *P<0.05, **P<0.01, ****P<0.0001.

HIV/HBV coinfected patients in our study had a rapid reduction of HBsAg level during the first 6 months, averaging 0.47 log_10_ IU/mL, and achieved a decline of 1.39 log_10_ IU/mL after five years of therapy. We investigated the effects of HBeAg positivity and baseline HBsAg levels on HBsAg decline. PWH with positive HBeAg results had significantly higher HBsAg levels at baseline and 6 months (**Figure 2A**). No difference was observed in the HBsAg decline between the HBeAg-positive and-negative groups (**Figure 2B**). Fifteen patients had HBsAg levels <1000 IU/mL at baseline and remained at a lower level during follow-up (**Figure 2C**). However, patients showed a similar trend in HBsAg decline, regardless of whether they had lower or higher HBsAg levels at baseline (**Figure 2D**).

Regarding the correlation between baseline CD4^+^ T cell counts and the HBsAg decline, 18 patients had a CD4^+^ T cell counts lower than 200 cell/mm^3^ and 33 patients had a count higher than 200 cell/mm^3^. Similar absolute HBsAg levels were observed in the patients with CD4^+^ T cells less than 200 cells/mm^3^ and those with CD4^+^ T cells more than 200 cells/mm^3^ (**Figure 2E**). However, patients with CD4^+^ T cells less than 200 cells/mm^3^ had a significantly greater HBsAg decline than those with CD4^+^ T cells more than 200 cells/mm^3^ (**Figure 2F**).

### 3.3 Serum sPD-1 profile and the association with HBsAg level

**Figure 3:**
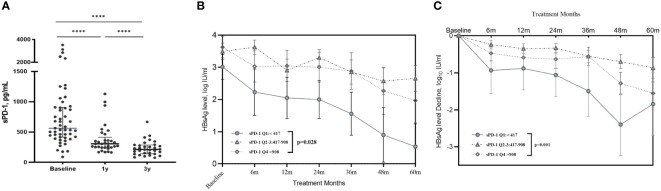
Serum sPD-1 profile and the association between sPD-1 with HBsAg decline. **(A)** The scatter dot plot shows the serum sPD-1 concentration in patients with HIV/HBV coinfection before and after treatment. Each dot represents a single patient. *P* values were determined using the Wilcoxon matched paired signed-rank test. **** *P*<0.0001. **(B)** The line graph shows the mean ± SEM of serum HBsAg level in coinfected patients stratified on baseline sPD-1 level. **(C)** The line graph shows the mean ± SEM of decline in serum HBsAg levels in coinfected patients stratified by baseline sPD-1 levels. *P*-values were determined using a linear mixed-effects model. ****P<0.0001.

Serum sPD-1 levels were measured to explore the profile of sPD-1 in HIV/HBV coinfection and its role in HBsAg decline. The baseline sPD-1 level in patients with HIV/HBV coinfection was 563 (interquartile range (IQR): 417–908) pg/ml, which decreased dramatically after receiving cART (P<0.001) (**Figure 3A**). The median sPD-1 level was 307 (IQR:244–506) pg/ml and 255 (IQR:202–439) pg/ml in the first and third years of treatment, respectively. In our study, patients with HIV/HBV with lower sPD-1 levels (Q1: <417 pg/mL) at baseline had lower HBsAg levels and greater HBsAg decline during follow-up (**Figures 3B, C**).

### 3.4 The clinical characteristics at baseline associated with HBsAg response

Patients with HIV/HBV coinfection were categorized into the following two groups to determine the factors related to early HBsAg decline in coinfected patients: the HBsAg decline response group (sAg-R) and the HBsAg non-response group (sAg-NR), as defined in the Methods (**Figure S1**). In this study, 17 (33.3%) participants achieved HBsAg response. Among them, five patients achieved HBsAg clearance at a median follow-up of 11 months(range: 6-51 months), and four patients experienced seroconversions. Their anti-HBs titers at the last visit ranged from 38.63 to >1,000 mIU/mL.

Baseline characteristics and differences are presented in **Table 2**. The sAg-R group had a relatively higher HBV-DNA load (5.88 log_10_ IU/mL vs. 2.28 log_10_ IU/mL, *P*=0.030) and a decreased count of CD4^+^ T cell at baseline than sAg-NR group (167 cell/mm3 vs. 257 cell/mm3, P=0.009). The sAg-R group was more likely to have CD4^+^ T cell counts below 200 cells/mm^3^ than the sAg-NR group, with a significant difference (58.8% vs. 23.5%, P=0.013). However, no significant difference was observed in the proportion of sPD-1 levels below Q1 (<417 pg/ml) between the sAg-R and sAg-NR groups (41.2% vs. 17.6%, P=0.069).

**Table 2:** Clinical characteristics of patients with or without HBsAg response.

Baseline characteristic	HBsAg response (n=17)	HBsAg non-response (n=34)	p-value
Age	35.8 (8.8)	34.9 (7.2)	0.412
Sex (% male)	16 (94.1)	31 (91.2)	1.000
HBeAg positive (%)	7 (41.2)	12 (35.3)	0.682
HBsAg level, log_10_ IU/mL	3.76 (1.85-4.86)	3.82 (3.11-4.15)	0.963
HBV DNA level, log10 IU/mL	5.88 (2.60-8.23)	2.28(1.50-5.54)	0.030
HBV DNA<20 IU/ml at 1 year (%)	7 (41.2)	7(20.6)	0.183
ALT(U/L)	26 (19-42)	24 (18-40)	0.761
AST(U/L)	27 (25-42)	25 (21-37)	0.153
Total bilirubin, μmol/L	11.8 (5.6)	12.8 (4.6)	0.332
Platelet count, x10^9^/L	177 (46)	180 (49)	0.785
APRI score	0.49(0.31-0.54)	0.37 (0.29-0.66)	0.314
FIB-4 score	1.53 (0.84-1.92)	1.03(0.74-1.73)	0.205
sPD-1	611 (353-1125)	544 (453-903)	0.704
sPD-1.Q1(< 417pg/mL)	7 (41.2)	6 (17.6)	0.069
HIV-RNA, log_10_ copies/mL	4.83 (2.76-5.12)	4.30 (3.50-4.83)	0.308
CD4^+^ T cell	167 (94)	257 (122)	0.009
CD4 T cell <200 cell/mm^3^	10 (58.8)	8 (23.5)	0.013
CD4/CD8 Ratio	0.27 (0.20)	0.32 (0.16)	0.325
TDF/TAF containing ART	16 (94.12)	26 (76.5)	0.241

Data are presented as mean(standard deviation), number of patients(percent) or median(Inter quartile range). Data analyzed by the t-test, Mann-Whitney U test or χ2 test.

HBeAg, hepatitis B envelope antigen; HBsAg, hepatitis B surface antigen; ALT, alanine aminotransferase; AST, aspartate aminotransferase; APRI, AST to Platelet Ratio index; FBI-4, Fibrosis-4; ART, antiretroviral therapy; sPD-1, soluble programmed death-1.

Logistic regression analyses were performed to evaluate the association between the clinical characteristics and HBsAg response (**Table 3**). Multivariate logistic Model 1 included HBV DNA (log_10_ IU/mL), CD4^+^ T cell counts <200 cell/mm^3^ (categorically coded), and sPD-1: Q1<417 pg/mL or not (categorically coded) as covariates to assess their influence on HBsAg response. Baseline HBV-DNA (odds ratio (OR)=1.287, P=0.044), CD4^+^ T cell count below 200 cells/mm^3^(OR=6.633, P=0.012), and low sPD-1 levels (OR=5.389, P=0.038) were risk factors for HBsAg decline. As TDF-based ART regimens have been reported to facilitate HBsAg decline (Audsley et al., 2020), TDF/TAF use was included as a covariate in Model 2. The Model 2 results showed that TDF/TAF use were significantly associated with HBsAg response(OR=38.441, P=0.011). CD4^+^ T cells and sPD-1 remained significant in Model 2; however, HBV DNA was no longer present.

**Table 3:** Univariate and multivariate logistic regression analysis with the determinants of HBsAg response.

Variables	Univariate		Multivariate (Model 1)		Multivariate (Model 2)	
	OR (95% CI)	*p* value	OR (95% CI)	*p* value	OR (95% CI)	*p* value
Age	1.017 (0.943-1.097)	0.668				
Gender (female)	1.548 (0.149-16.110)	0.714				
HBeAg status	1.283 (0.389-4.239)	0.682				
HBsAg level	0.841 (0.533-1.327)	0.457				
HBV DNA	1.240 (1.007-1.526)	0.042	1.287 (1.007-1.646)	0.044		0.205
ALT	1.003 (0.996-1.010)	0.381				
HIV-RNA	1.128 (0.735-1.732)	0.582				
CD4 count	0.993 (0.987-0.999)	0.015				
CD4 count<200	4.463 (1.331-16.195)	0.016	6.633 (1.515-29.034)	0.012	18.289 (3.110-107.549)	0.001
CD4/CD8 ratio	0.171 (0.005-5.905)	0.329				
sPD-1<Q1	3.267 (0.883-12.080)	0.076	5.389 (1.097-26.464)	0.038	8.740 (1.577-48.436)	0.013
TDF/TAF use	4.923 (0.562-43.123)	0.150			38.441 (2.271-650.674)	0.011

The significant variables (P < 0.10) in univariate analysis were included in multivariate logistic regression model 1.

Model 2 was fitted on TDF/TAF containing cART and variables with difference (P < 0.10).

sPD-1, soluble programmed death-1; OR, odd ratio; CI, confidence interval.

### 3.5 ALT abnormalization and immune activation in HBsAg response group during treatment

**Figure 4:**
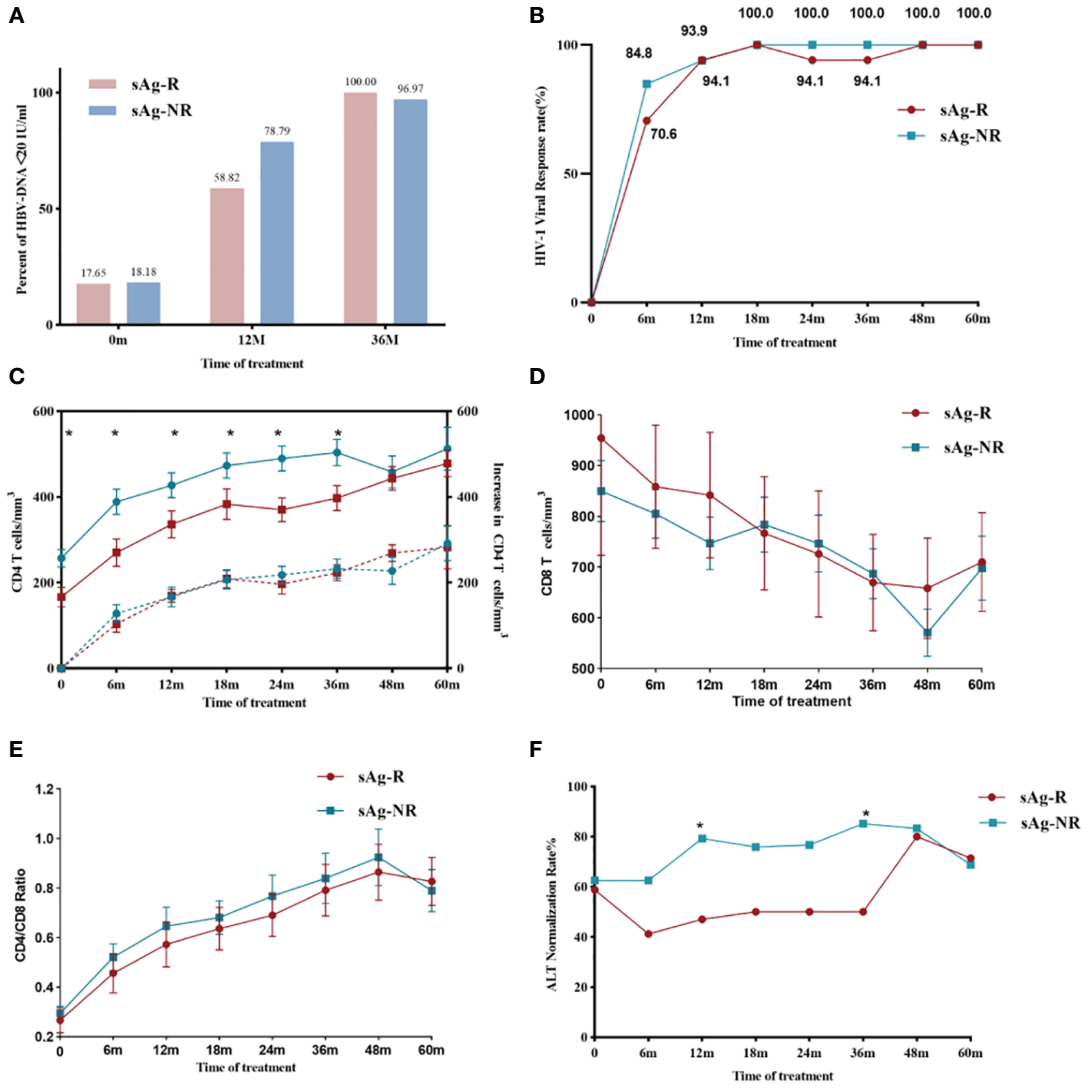
HBV and HIV viral and immunological responses during treatment. **(A)** The column chart shows HBV viral control in the sAg-R (red column) and the sAg-NR groups (blue column) during treatment. **(B)** The line graph shows the HIV viral control rate in patients in the sAg-R (red line) and sAg-NR groups (blue line) during treatment. **(C)** The line graph depicts the mean CD4^+^ T cell counts (solid line and left y-axis) and mean increased CD4^+^ T cell counts (dashed line and left y-axis) of the sAg-R group (red line) and sAg-NR group (blue line) during the follow-up. Error bars indicate SEM. **(D)** Line graph depicting mean CD8^+^ T cell counts and **(E)** mean CD4^+^/CD8^+^ ratio in patients with HIV/HBV coinfection during follow-up. Error bars indicate SEM. **(F)** Line graph showing the ALT normalization rate in the sAg-R (red column) and sAg-NR groups (blue column) during follow-up. sAg-R, HBsAg response group. sAg-NR, HBsAg non-response group. *P<0.05.

We analyzed long-term clinical features characterized by HBsAg responses. During follow-up, no significant difference was found in HBV or HIV viral control between the sAg-R and sAg-NR groups (**Figures 4A, B**). The sAg-R group had a lower CD4^+^ T cell count at baseline and during follow-up. However, no difference was found in the increase in CD4^+^ T cell counts between the two groups (**Figure 4C**) or in the CD8^+^ T cell counts and CD4^+^/CD8^+^ ratio (**Figures 4D, E**). However, the rate of ALT normalization in the sAg-R group was higher than that in the sAg-NR group (**Figure 4F**). A similar trend was observed for fibrosis scores, as shown in **Figure S2**.

**Figure 5:**
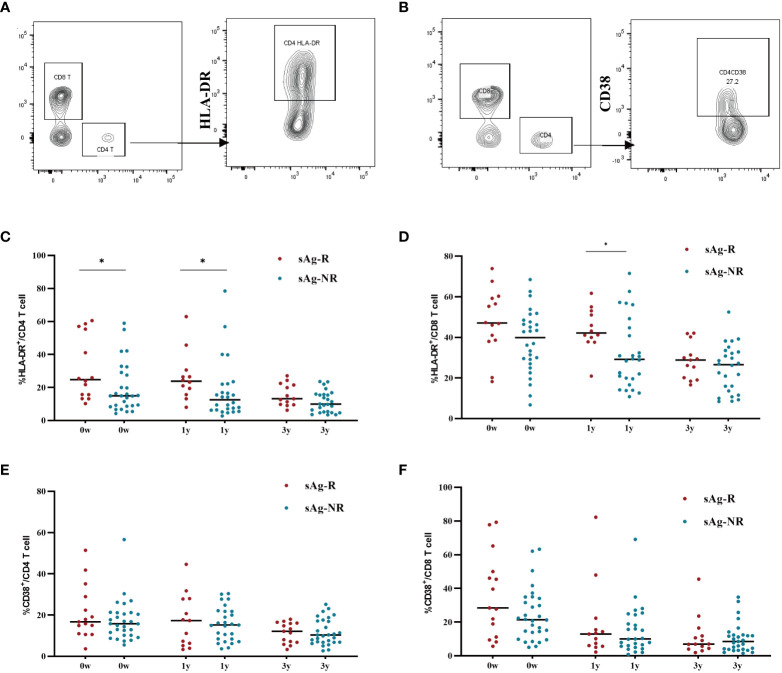
The expression of HLA-DR and CD38 on CD4+ and CD8+ cell. **(A, B)** Gating strategy for measuring HLA-DR and CD38 expression in T cells. **(C, D)** Scatter dot plots showing the expression of HLA-DR in CD4^+^ and CD8^+^ T cells with median values. Each plot represents a single patient. **(E, F)** Scatter-dot plots depicting the expression of CD38 on CD4^+^ and CD8^+^ T cells with median values. Each plot represents a single patient. sAg-R, HBsAg response group. sAg-NR, HBsAg non-response group. The Mann–Whitney U test was used to compare data between sAg.R and sAg-NR, *P<0.05.

To evaluate immune activation in participants with an HBsAg response, we also assessed HLA-DR and CD38 expression on T cells. Gating strategies for measuring HLA-DR and CD38 expression were shown in **Figures 5A, B**. As shown in **Figures 5C, D**, PWH with HBsAg response (sAg-R) had significantly higher expression of HLA-DR on CD4^+^ and CD8^+^ T cells than those with HBsAg non-response (sAg-NR) during treatment. The expression of CD38 on CD8^+^ T cells between the two groups showed a similar trend but was not significantly different (**Figures 5E, F**). Overall, these data demonstrate that patients with HBsAg response had a higher level of inflammation and immune activation of T cells than those in the sAg-NR group at baseline and longitudinally.

## 4 Discussion

We investigated the HBsAg decline kinetics in patients with HIV/HBV coinfection undergoing long-term cART. We also analyzed clinical variables associated with HBsAg response (more than 0.5 log10 decline in the first 6 months) in HIV/HBV coinfection. Our findings suggest that lower CD4^+^ T cell counts and sPD-1 levels are associated with rapid HBsAg decline after the initiation of cART. During longitudinal follow-up, we observed high rates of ALT abnormalities and upregulated HLA-DR expression on T cells in PWH with HBsAg response, suggesting that inflammation and immune activation may be related to HBsAg clearance in HIV/HBV coinfection.

HBsAg seroclearance is an essential standard for functional cure evaluation and is an ideal endpoint for treating HBV infection (Lok et al., 2017; Cornberg et al., 2020). However, HBsAg seroclearance rarely occurs in patients with CHB receiving NAs therapy. Recently, a large cohort study showed that HBsAg seroclearance occurred in 1.69% of the patients receiving Entecavir and 1.34% of patients receiving TDF (Hsu et al., 2022). Interestingly, several studies have reported that HBsAg seroclearance in patients with HIV/HBV coinfection is significantly higher than that in those with HBV monoinfection, ranging from 3.7% to 34.5% (Jiang et al., 2019; Audsley et al., 2020; van Bremen et al., 2020; Boyd et al., 2021; Yoshikawa et al., 2021). In this study, we observed that five patients with HIV/HBV coinfection achieved HBsAg seroclearance. The HBsAg seroclearance rate in the HIV/HBV population varies depending on the study population, HBV genotype, and follow-up duration (Yang et al., 2020; Yoshikawa et al., 2021).

We found that lower baseline CD4^+^ T cell counts were significantly correlated with a rapid decline in HBsAg levels. Previous studies have reported that CD4^+^ T cell recovery is associated with HBsAg loss (Zoutendijk et al., 2012). However, our study did not identify any association between an increase in CD4^+^ T cell counts and a HBsAg response. Similarly, Chihota et al. reported that a lower baseline CD4+ T cell count, rather than an increase in CD4+ T cells, was related to the HBsAg response (Chihota et al., 2020). It is possible that the recovery of more HBV naive T cells after cART contributes to this phenomenon. Furthermore, patients with higher baseline HBV DNA levels were also more susceptible to an HBsAg response. Consistent with our results, a systematic review showed that patients who presented with lower baseline CD4^+^ T cell counts and higher levels of HBV DNA had a higher likelihood of experiencing HBsAg seroclearance. (Jiang et al., 2019). Another interesting finding was that abnormal alanine aminotransferase levels occurred frequently during therapy in the HBsAg response group. In HBV mono-infection therapy, fluctuations in liver enzymes usually indicate anti-HBV immune response and could predict HBsAg loss (Sonneveld et al., 2013; Wong et al., 2018; Ghany et al., 2020; Yao et al., 2022). ALT flare also has similar prognostic significance in coinfected individuals (Yoshikawa et al., 2021; Iannetta et al., 2022).

Increased immune activation was observed in the HBsAg-responsive group. Regarding the low CD4 T cell level and high ALT flare in the HBsAg response group, it is reasonable to speculate that HIV-induced host immune disturbances and activation break down the immunotolerance of HBV and begin to clear the virus in the presence of efficient cART. In chronic HBV infection, the host immune system usually ignores the virus due to immune tolerance, particularly in individuals infected during childhood, which is common in China. Immune disturbances induced by coinfection with HIV may disrupt immunotolerance. The immune system recognizes and initiates viral clearance with the help of effective cART (3TC+ TDF-containing regimens), resulting in a rapid HBsAg decline and liver inflammation, as evidenced by higher ALT abnormalization and immune activation in the sAg-R group. This phenomenon may be more pronounced in coinfected patients with low CD4^+^ cell counts due to more intense immune disturbances. Immune reconstitution-induced hepatic flares may be an extreme manifestation of this phenomenon and are associated with an extremely high rate of HBsAg loss among patients with HIV/HBV coinfection (Wong et al., 2018; Ghany et al., 2020; Yoshikawa et al., 2021; Iannetta et al., 2022). However, further studies on the immunological mechanisms are warranted to validate this hypothesis.

Our study represents the first investigation into sPD-1 profiles in HIV/HBV coinfected individuals and provides novel insights into the role of sPD-1 in this context. Our findings indicate that sPD-1 levels were elevated at baseline and subsequently decreased during long-term antiretroviral therapy. During HIV infection, sPD-1 levels are closely associated with PD-1 expression on T cell surfaces (Zilber et al., 2019). Therefore, reduced sPD-1 levels suggest decreased immune exhaustion and may contribute to viral control. Interestingly, our study revealed that lower baseline sPD-1 levels were independently associated with greater declines in HBsAg levels during cART. This inverse association is consistent with previous studies of HBV mono-infection, which demonstrated that lower sPD-1 levels are an effective predictor of HBeAg seroclearance (Xia et al., 2020; Chu et al., 2022) and spontaneous HBsAg seroclearance in inactive CHB patients with undetectable HBV DNA (Hu et al., 2022). Furthermore, sPD-1 levels were not correlated with CD4^+^ T cell levels (r=0.0985, P=0.4915) in our study, indicating the effects of sPD-1 and CD4^+^ T cells on HBsAg decline are independent of each other. It also suggests that sPD-1 levels in HIV/HBV coinfection are not only influenced by the HIV-induced immune mechanism, but also by other factors. Given the potential benefits of low sPD-1 levels for HBsAg decline, it is important to explore the possibility of reducing sPD-1 generation through immunotherapy, such as PD-1/PD-L1 inhibitor. Nevertheless, further investigations are required to fully confirm and elucidate the role of sPD-1 in HBsAg decline in more detail.

Our study had some limitations. First, the follow-up intervals were not stringent, which may have induced bias. Second, the sample size was relatively small, which limited the generalizability of our conclusions to other settings. Finally, overall, HBsAg seroclearance was insufficient; therefore, we could not evaluate the diagnostic performance of sPD-1 and CD4^+^ T cell counts for HBsAg seroclearance. Therefore, a well-designed large-cohort study should be conducted to validate our findings.

In conclusion, this exploratory study suggests that monitoring CD4^+^ T cell counts and sPD-1 levels may have implications for HBsAg decline in patients with HIV/HBV coinfection. Notably, patients who experience a significant decline in HBsAg exhibit higher liver inflammation and immune activation. Future exploration of the dynamic changes of the immune system after cART initiation could provide valuable insights for developing novel therapies and designing clinical trials of chronic HBV infection.

## Data availability statement

The original contributions presented in the study are included in the article/**Supplementary Material**. Further inquiries can be directed to the corresponding author.

## Ethics statement

The studies involving human participants were reviewed and approved by Institutional Review Board of PUMCH. The patients/participants provided their written informed consent to participate in this study.

## Author contributions

XDL, WC and TL contributed to the design, and interpretation of the results and drafts of the manuscript. XS, YL, XXL and WC contributed to regular clinic visits and data collection. XDL, LX, LL, XSL, YY, YW and YH performed experiments, statistical analyses, and validation. WC and TL were involved in the supervision, funding acquisition, and manuscript review. All authors contributed to the article and approved the submitted version.

## Supplementary material

Supplementary Figure 1 HBsAg decline in HIV/HBV coinfected patients with or without HBsAg response. **(A)**The line graph shows mean± SEM of serum HBsAg level and **(B)** HBsAg decline in patients with HIV/HBV coinfected with HBsAg response or non-response. sAg-R, HBsAg response group. sAg-NR, HBsAg non-response group. **P*<0.05,***P*<0.01,****P*<0.001.

Supplementary Figure 2 Longitudinal liver fibrosis score in patients with HIV/HBV coinfection during treatment. **(A)** Scatter dot plots depicting the APRI and **(B)** FBI-4 scores of patients in the sAg-R (red dot) and sAg-NR (blue dot) groups during follow-up, with median values. The Mann–Whitney U test was used to compare data between sAg.R and sAg-NR. APRI, AST to Platelet ratio index; FBI-4, Fibrosis-4; sAg-R, HBsAg response group. sAg-NR, HBsAg non-response group. **P*<0.05.
