# Supplementary figures and images for: CD4+ T cell counts and soluble programmed death-1 at baseline correlated with hepatitis B surface antigen decline in HIV/HBV coinfection during combined antiretroviral therapy
Source: Front Cell Infect Microbiol. 2023 May 3;13:1178788. doi: 10.3389/fcimb.2023.1178788 (PMC10189149; doi:10.3389/fcimb.2023.1178788)

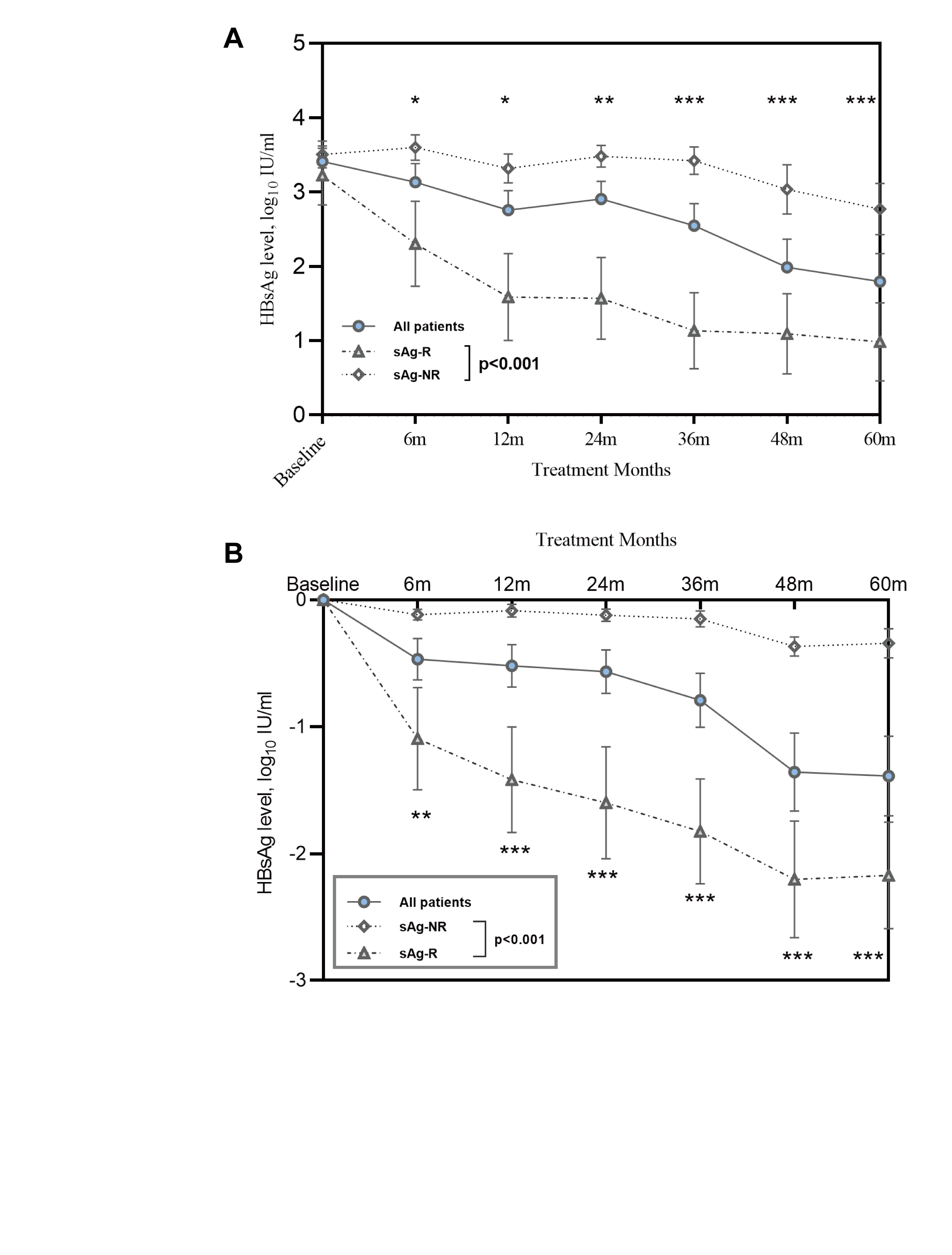

Supplement: Supplementary Figure 1 — HBsAg decline in HIV/HBV coinfected patients with or without HBsAg response. (A)The line graph shows mean± SEM of serum HBsAg level and (B) HBsAg decline in patients with HIV/HBV coinfected with HBsAg response or non-response. sAg-R, HBsAg response group. sAg-NR, HBsAg non-response group. *P<0.05,**P<0.01,***P<0.001. [file Image_1.tif]

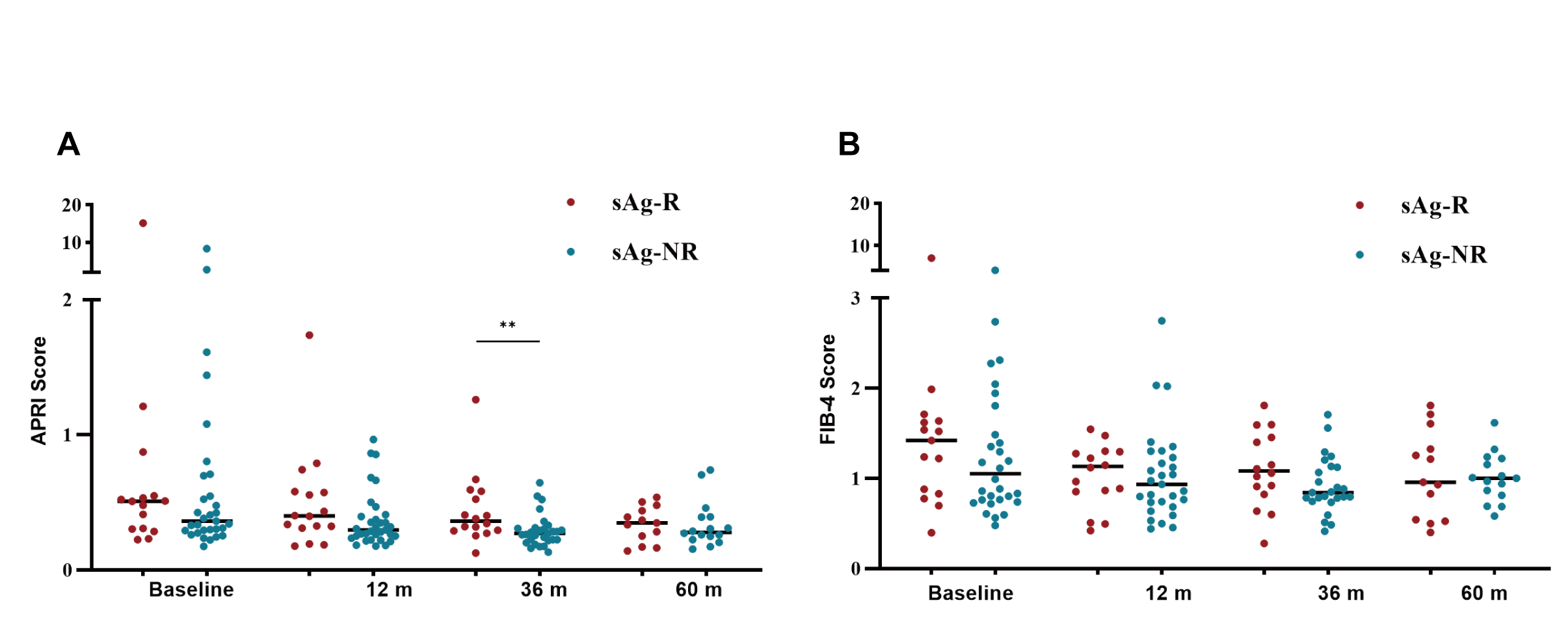

Supplement: Supplementary Figure 2 — Longitudinal liver fibrosis score in patients with HIV/HBV coinfection during treatment. (A) Scatter dot plots depicting the APRI and (B) FBI-4 scores of patients in the sAg-R (red dot) and sAg-NR (blue dot) groups during follow-up, with median values. The Mann–Whitney U test was used to compare data between sAg.R and sAg-NR. APRI, AST to Platelet ratio index; FBI-4, Fibrosis-4; sAg-R, HBsAg response group. sAg-NR, HBsAg non-response group. *P<0.05. [file Image_2.tif]
